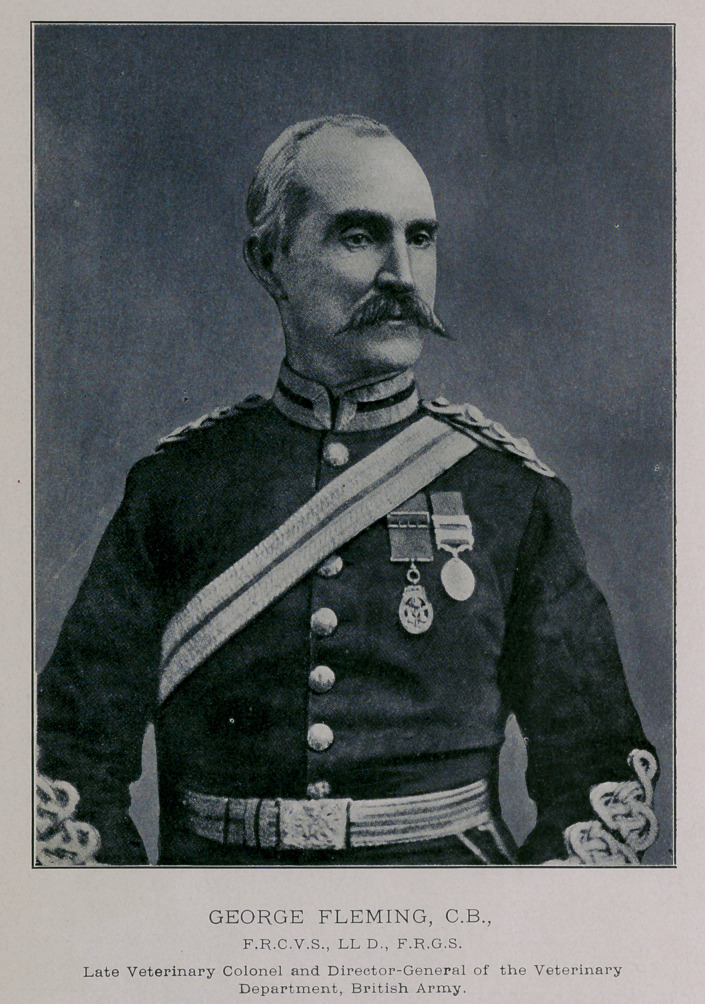# Necrology

**Published:** 1901-05

**Authors:** 


GEORGE FLEMING, C.B.,

F.R.C.V.S., LL D., F.R.G.S.

Late Veterinary Colonel and Director-General of the Veterinary Department, British Army.

State has divided the ranks of the profession. There is much to be said for and against both sides in the wordy conflict now going on, but we believe the greater danger is to the profession as a whole, and we will not publish any further letters on the subject which are so personal in character. We strongly urge that the profession get together and endeavor to correct the evils complained of.

### NECROLOGY.

### George Fleming, C.B., LL.D., F.R.C.V.S.,

LATE VETERINARY COLONEL AND DIRECTOR-GENERAL OF THE VETERINARY DEPARTMENT, BRITISH ARMY.

George Fleming was born at Glasgow on March 11, 1833. Early in life he accompanied his father to Manchester, where both were employed in the farrier shop of a veterinary surgeon. From this he entered the service of the late Mr. John Lawson. Mr. Lawson was one of those rare men who are able to recognize talent and willing to assist its development. To this fact was due Fleming's first employment as veterinary assistant and pupil and his being sent to Dick's College in Edinburgh. Fleming is said by those who then knew him to have been fond of books and distinctly ambitious. His industry was equal to his talents, and his college career was a marked success. He obtained medals for chemistry, materia medica, and anatomy, for essays, and the Fitz-Wygram prize for practical knowledge.

In 1855 he obtained the certificate of the Highland and Agricultural Society, which was then acknowledged as a veterinary diploma. Toward the end of the year he entered the army and served as a veterinary officer in the Crimea until the termination of the war.

In 1859 he volunteered to serve in the expedition to North China, and was present at the capture of the Taku forts and the surrender of Pekin. He remained in China until 1861. While there he and a friend undertook an expedition beyond the Great Wall, a description of which formed the subject of his first published book—*Travels on Horseback in Manchu Tartary*.

In 1863 he read papers before the British Association at Newcastle-on-Tyne on the "Geography of North China" and on the "Ethnology of that Country."

In 1866 he received the diploma of the Royal College of Veterinary Surgeons. The mere passing the examination was of course a simple matter, but it was an act of some moment to the veterinary profession. At that time there was extreme rivalry and much feeling between the holders of the Highland and Agricultural Society's certificate and the diplomas of the R. C. V. S. For some years attempts had been made to bring about union and harmony. In 1866 a number of certificate holders had been induced to join the R. C. V. S., and the college had made special arrangements for their admission. It was not till some years later that the union was effected, but Fleming and those who took the first step may be credited with having done much toward the final establishment of one portal to the veterinary profession.

In 1867 Fleming served with the army in Syria and Egypt. On his return he spent some years with the Royal Engineers at Chatham.

In 1879 he was appointed Inspecting Veterinary Surgeon at the War Office, and in 1883 succeeded to the office of Principal Veterinary Surgeon to the Forces. In 1887 he was made a Companion to the Bath (C.B.), and in 1890 retired from the army.

During these long years of regimental and departmental duties Fleming found time for the production of an enormous amount of literary work and for constant activity in veterinary politics. His contributions to veterinary periodicals would fill pages only to enumerate. His larger works are a tolerably long list, as follows:

1866, *Vivisection: Is it Necessary or Justifiable.* 1869, *Horseshoes and Horseshoeing; their Origin, History,* etc. 1871, *Animal Plagues; their History, Nature, and Prevention,* vol. i. 1872, *Practical Horseshoeing,* a prize essay. 1872, *Rabies and Hydrophobia,* 405 pages. 1873, *The Comparative Anatomy of the Domesticated Animals* (a translation of Chauveau's French work). 1875, *A Manual of Veterinary Sanitary Science,* 2 vols. 1878, *A Text-book of Veterinary Obstetrics,* 759 pages. 1882, *Animal Plagues,* vol. ii. 1883, *The Influence of Heredity and Contagion in the Propagation of Tuberculosis.* 1884, *Operative Veterinary Surgery,* vol. i. 1886, *The Practical Horsekeeper.* 1894, *The Parasites and Parasitical Diseases of the Domesticated Animals,* 800 pages.

In addition to these technical volumes Fleming contributed largely to *The Encyclopædia Britannica, Lancet, British Medical Journal, Medical Press and Circular, Veterinarian, Veterinary Journal, Live-stock Journal, The Journal of the Royal Agricultural*

*Society*, *The Journal of the Society of Arts*, *The Nineteenth Century*, and many other periodicals, foreign and colonial. In 1876 he established *The Veterinary Journal*, and carried it on with conspicuous ability and energy until quite recently. Last year he presented his technical library of close upon 800 volumes to the R.C.V.S., a gift so far unequalled in our annals.

In the business and politics of the R.C.V.S. Fleming showed the same strenuous activity and earnestness that marked the rest of his career. Joining the Body Corporate in 1866, he was elected a Vice-President next year and a Member of Council in 1868. He filled the office of President in 1880 and gave such satisfaction that the Council retained him in that position until 1884. In 1886, on the retirement of the elected president, Fleming was again called to preside. Since 1876 to the present time he has been a member of the Court of Examiners. His regular table was anatomy, but at various times he has officiated at the tables for materia medica, pathology, and morbid anatomy. He was also an examiner for the Fellowship.

During the thirty and odd years Fleming was so closely associated with the governing body of the veterinary profession all the real reforms which have marked our progress have taken place, and he has left his impress upon all but the latest. He assisted in framing and obtaining the earlier supplementary charters. He helped the union of the Scotch and English branches of the profession. He worked hard to raise the standard of education and to increase the curriculum of the schools, but his greatest achievement was in 1881, when he was the chief instrument in obtaining the "Veterinary Surgeons' Act." This Act gives us our title, prevents its assumption by unqualified persons, and permits the Council to remove from our register undesirable members who have forfeited the respect of decent people. When the bill was before the House, Fleming attended daily for five weeks, and the Right Hon. Joseph Mundella told at a public dinner how, when he was a Cabinet Minister in 1881, a veterinary surgeon was always haunting him in the interests of the bill, and how one night, when an accidental pause in Parliamentary proceedings offered a chance, he was pressed and driven to push in the bill, and so it passed, though strongly opposed. The man that looked so closely after our interests was George Fleming, and we cannot be too grateful.

Another direction in which he played a part for good was the interest he took in local veterinary societies—some of which he helped to establish and all of which he assisted by taking part in their proceedings. We fancy there is not a society of which he was not an honorary member at the time of his death. He was the first President of the National Veterinary Association and an active member for many years.

Outside of the veterinary profession he found further scope for his energies. He was an honorary life-member of the Royal Society for the Prevention of Cruelty to Animals, a Fellow of the Society of Arts, honorary life-member of the Royal Agricultural Society, Fellow of the Anthropological Society, Fellow of the Royal Geographical Society, a Governor of the Royal Veterinary College, Member of the Pathological Society, Member of the Epidemiological Society, Member of the Royal United Service Institution, Member of Council of the British Institute of Preventive Medicine.

Foreign and Colonial bodies recognized his position by making him Honorary Foreign Correspondent of the Royal Academy of Medicine of Belgium ; Foreign Associate of the National and Central Society of Veterinary Medicine, Paris ; Honorary Member of the Veterinary Medical Society, Frankfort ; Corresponding Member of the Royal National Society of Veterinary Medicine, Italy ; Honorary Member of the United States Veterinary Medical Association ; Honorary Member of the Veterinary Association of Montreal, Canada; Honorary Member of the Australian Veterinary Association.

In 1883 the University of Glasgow conferred upon him the degree of Doctor of Laws (LL.D.) Honoris Causa. Twice presentations were made—one of plate and money, and one of his portrait, which adorns the Board Room of the R. C. V. S.

His career has been a wonderful one—from forge-boy to Director-General of the Veterinary Department of the British Army ! Self-taught, he mastered several modern languages, wrote a dozen big books, scores of essays, and thousands of articles. While carrying out successfully his military duties he has assisted to reform his profession and direct its politics. His industry has been phenomenal, and through all this stress and strain he has retained the respect and gratitude of the profession.

Dr. Fleming was ambitious, he was impulsive, and somewhat dogmatic. As a controversialist he did not shine.

In private life he was a genial, interesting, and amusing friend. Possessed of great vitality and a strong sense of humor, he always took a leading part in any company. He had travelled far and observed acutely; he was well read, and possessed a remarkable memory—traits which made him a brilliant conversationalist. His personality will long be remembered and his work will never be forgotten.

## Figures and Tables

**Figure f1:**